# Preparation, Characterization, and Anti-Colitis Activity of Low-Viscosity EDTA-Soluble Polysaccharides from Almond Gum

**DOI:** 10.3390/foods15061103

**Published:** 2026-03-21

**Authors:** Munisa Dilixiati, Zumrat Abudureyim, Nuermaimaiti Abudukelimu, Ahmidin Wali, Yanhua Gao, Abulimiti Yili

**Affiliations:** 1 State Key Laboratory Basis of Xinjiang Indigenous Medicinal Plants Resource Utilization and Key Laboratory of Plants Resources and Chemistry of Arid Zone, Xinjiang Technical Institute of Physics and Chemistry, Chinese Academy of Sciences, Urumqi 830011, China; mns21@ms.xjb.ac.cn (M.D.); nuratux@ms.xjb.ac.cn (N.A.); ahmidin@ms.xjb.ac.cn (A.W.); gaoyh@ms.xjb.ac.cn (Y.G.); 2 University of Chinese Academy of Sciences, Beijing 100039, China; 3 Morphological Center, School of Basic Medical Sciences, Xinjiang Medical University, Urumqi 830011, China; zumrat1208@xjmu.edu.cn

**Keywords:** *Amygdalus communis* L. gum, Polysaccharide, response surface design, ethylenediaminetetraacetic acid, ulcerative colitis, gut microbiota

## Abstract

Almond gum is a resource-rich natural polysaccharide; however, its high viscosity and low solubility severely limit industrial applications in separation, purification, and functional development. This study aimed to overcome these bottlenecks by optimizing an ethylenediaminetetraacetic acid (EDTA) preparation process and evaluating its protective efficacy against colitis. Using response surface methodology, optimal conditions were identified (1% EDTA, 3 h reaction, 10 h extraction), resulting in a modified polysaccharide (EAGP) with significantly reduced viscosity (from 640.8 to 238.7 mPa·s). SEM-EDX confirmed that EDTA efficiently removed cross-linking metal ions (K, Ca, Mg), creating a porous structure that facilitates purification. The purified fraction, EAGP-W1, was characterized as an arabinogalactan primarily composed of galactose (40.51%) and arabinose (38.38%). In vivo experiments demonstrated that EAGP-W1 significantly alleviated DSS-induced colitis, reducing colonic shortening and histopathological damage (*p* < 0.05). Mechanistically, EAGP-W1 reshaped the gut microbiota by downregulating pro-inflammatory genera and upregulating probiotics (*p* < 0.05). This shift promoted the production of short-chain fatty acids (SCFAs) (*p* < 0.05), thereby repairing the intestinal barrier and suppressing inflammation. Overall, this study establishes an efficient EDTA-based strategy for almond gum processing and elucidates its anti-inflammatory mechanism through the “microbiota–metabolite–barrier” axis, providing a theoretical basis for its development as a high-value functional food for gut health.

## 1. Introduction

Almonds (*Amygdalus communis* L.), a highly economically valuable nut tree species in the Rosaceae family, are cultivated on a large scale across numerous regions worldwide. Particularly in China’s Xinjiang region, where suitable climate and soil conditions prevail; the species boasts extensive planting areas and stable yields [1]. It offers multifaceted economic benefits: not only are its nuts highly prized as a healthy food by consumers, but the natural gum secreted from its tree trunk when physically damaged or environmentally stressed is a valuable resource rich in functional components such as polysaccharides and polyphenols [2,3]. These natural gum polysaccharides are not only abundant, easily extractable, and cost-effective but also safe, non-toxic, and renewable [4,5]. Its main component of almond gum is carbohydrates (92.36%), consisting primarily of arabinose (46.8%), galactose (35.5%), xylose (10.9%), and uronic acid (6.0%), indicating that it has an arabinogalactan structure [6]. Previous studies have reported that almond gum polysaccharides (AGPs) and almond gum hemicellulose (AGH), extracted from almond gum, exhibit antimicrobial properties and potent angiotensin-converting enzyme inhibitory activity (75.81% and 90.86%, respectively). These findings suggest that AGP and AGH hold promise for applications in both the food and non-food industries [7]. In addition, almond gum polysaccharides exhibit strong antioxidant activity and potential prebiotic properties [8,9]; furthermore, they can be used in the food industry as foaming agents, stabilizers, and emulsifiers [10]. New polysaccharide resources, such as almond gum, provide high-quality raw materials for the development of natural polymers, creating significant opportunities to extend the almond industry chain and enhance overall industrial benefits.

Given that natural gum is a complex polysaccharide, systematic research into the physicochemical properties and biological activities of almond gum polysaccharides relies on polysaccharide chemistry. A comprehensive study of polysaccharides involves establishing efficient protocols ranging from extraction and separation to purification; obtaining homogeneous fractions with low polydispersity through systematic purification is a prerequisite for elucidating their structure–activity relationships. In recent years, various extracts of almond gum polysaccharides have been explored for their biological activities [3,7,11,12]. However, systematic studies covering the full pipeline from extraction to purification remain scarce. The critical bottleneck is that polysaccharides obtained directly via aqueous extraction exhibit exceptionally high viscosity and low solubility, which severely hinders subsequent chromatographic purification and physicochemical characterization. Although conventional methods (e.g., hot water, ultrasound, microwave, or enzymatic assistance) can improve yield, they often fail to specifically disrupt the cross-linked network mediated by divalent cations (Ca^2+^, Mg^2+^) inherent to the gum structure, leaving the viscosity issue unresolved. In this context, ethylenediaminetetraacetic acid (C_10_H_16_N_2_O_8_, abbreviated as EDTA) offers a mechanistically distinct solution. As a potent metal ion chelator capable of forming stable, five-membered ring complexes [13], EDTA can precisely remove these cross-linking ions under mild conditions, thereby deconstructing the macromolecular network, reducing viscosity, and enhancing solubility. This approach not only facilitates polysaccharide release but also prevents metal-catalyzed degradation or impurity formation during extraction [14,15]. The success of EDTA-assisted extraction in isolating novel active polysaccharides from ginseng further validates its potential [16]. Therefore, we hypothesize that introducing an EDTA-based extraction strategy could overcome the purification barriers posed by the high viscosity of almond gum polysaccharides. To maximize the efficiency of this process, Response Surface Methodology (RSM) based on a Box–Behnken design (BBD) was employed to optimize extraction parameters [17,18]. This study aims to establish a systematic EDTA-assisted protocol for the extraction, separation, and purification of almond gum polysaccharides, thereby providing a foundation for subsequent investigations into their fine structures and biological functions.

Beyond extraction optimization, the biological potential of gum polysaccharides in intestinal health has gained increasing attention. Dextran sulfate sodium (DSS)-induced colitis is a widely recognized animal model for studying human ulcerative colitis, as it effectively simulates clinical symptoms such as mucosal erosion, inflammatory cell infiltration, and intestinal barrier dysfunction [19]. The therapeutic efficacy of polysaccharides is intricately linked to their specific structure–activity relationships. Structural features—such as monosaccharide composition, molecular weight, glycosidic linkages, and spatial conformation—strictly dictate their prebiotic and anti-inflammatory potentials [20]. Notably, almond gum polysaccharides are rich in arabinose and galactose, forming a highly branched arabinogalactan backbone. This specific structural feature is highly resistant to digestion in the upper gastrointestinal tract, allowing it to reach the colon intact, where it serves as a premium fermentable substrate for targeted beneficial gut microbiota (e.g., Bifidobacterium and Lactobacillus) [21,22]. Targeted fermentation of these arabinogalactan structures significantly increases the production of short-chain fatty acids (SCFAs), such as butyrate and acetate, which are crucial for maintaining physical barrier integrity and suppressing the release of pro-inflammatory cytokines [23]. Furthermore, the presence of uronic acid in almond gum polysaccharides may provide additional mucosal adhesion and reactive oxygen species (ROS) scavenging capabilities. Therefore, elucidating the specific structural characteristics of almond gum polysaccharides is vital for understanding its underlying prebiotic mechanisms and its protective effects against DSS-induced intestinal damage.

To bridge the gap between extraction technology and biomedical application, the present study aims to establish a comprehensive framework for the preparation and functional evaluation of almond gum polysaccharides. The primary objective was to optimize an EDTA-assisted extraction process using Response Surface Methodology (RSM), thereby effectively overcoming the inherent high-viscosity bottleneck. Building upon this optimized protocol, we systematically characterized the physicochemical properties and structural features of the purified almond gum polysaccharides to delineate their molecular profile. Furthermore, to explicitly link these structural characteristics with their biological potential, we evaluated the protective efficacy of almond gum polysaccharides against DSS-induced colitis in a murine model. Particular emphasis was placed on elucidating the underlying prebiotic mechanisms, specifically on modulating gut microbiota composition and enhancing short-chain fatty acid (SCFA) production. Ultimately, these integrated findings are expected to provide robust methodological references and theoretical insights into the structure–activity relationships of almond gum polysaccharides, thereby accelerating their high-value utilization in functional foods and biomedical therapeutics.

## 2. Materials and Methods

### 2.1. Materials

Almond gum samples were obtained as exudates from *Amygdalus communis* L. trees in the Kashgar Region of Xinjiang, China. After collection, the tree gum was ground into a powder, sieved to remove impurities, and then stored in a sealed container in a dry environment for subsequent experiments.

Dialysis membranes were produced by BioSharp (Hefei, China). Monosaccharide standards were obtained from Sigma-Aldrich (St. Louis, MO, USA). Ethylenediaminetetraacetic acid, Huazhong Huiwei Chemical Reagent Co., Ltd. (Wuhan, China); DEAE Sepharose Fast Flow and Superdex columns were obtained from GE Healthcare (AB, Uppsala, Sweden). Dextran sulfate sodium (DSS; molecular weight: 36,000–50,000, MP Biomedicals (Santa Ana, CA, USA)). All chemicals used in the study were of AR Grade.

### 2.2. Extraction and Preparation of AGP

The collected almond gum was naturally air-dried, pulverized, and sieved to obtain a fine gum powder. A 2% (*w*/*v*) aqueous solution of the gum powder was prepared and subjected to magnetic stirring at room temperature for 12 h. Subsequently, the mixture was centrifuged at 5000 rpm for 10 min. The resulting supernatant was collected and slowly added to four volumes of anhydrous ethanol under continuous stirring. To ensure complete precipitation, the mixture was stored at 4 °C for 12 h. The precipitate was then recovered by centrifugation and filtration. To remove pigments, the collected precipitate was washed three times with anhydrous ethanol and subsequently redissolved in deionized water. Finally, the solution was lyophilized to obtain the crude almond gum polysaccharide (AGP).

### 2.3. Preparation of EAGP

The collected almond gum was naturally air-dried, pulverized, and sieved to obtain gum powder. Prepare a 2% aqueous solution of almond gum for extraction. EDTA was added according to the experimental design ratio, after which the reaction was stirred for the set reaction time, and the mixture was centrifuged at 5000 rpm for 10 min. The supernatant was collected and mixed with fourfold the volume of anhydrous ethanol, stirring continuously until complete precipitation occurred. Place in a 4 °C refrigerator for 12 h, centrifuge and filter. Collect the precipitate, redissolve in deionized water, and place in a dialysis bag for 24 h of dialysis. Freeze-dry to obtain crude polysaccharide (EAGP).

### 2.4. Single-Factor Experiment

Reference [24] were reviewed and slightly adjusted. A (EDTA concentration: 0.5%, 1%, 1.5%, 2%, 2.5%), B (reaction time: 1, 2, 3, 4, and 5 h), and C (extraction time: 2, 4, 8, 10, and 12 h) were selected as single-factor designs to determine the preliminary range of preparation factors. The dependent variable was measured as follows: For each single-factor experiment, the supernatant after centrifugation was diluted fivefold, and the absorbance at 490 nm was determined using the phenol–sulfuric acid method. The absorbance measured by this method is directly proportional to the sample’s sugar concentration; higher absorbance indicates higher sugar content.

### 2.5. Response-Surface Optimization Experiment

#### 2.5.1. Response-Surface Methodology Experimental Design

Building upon single-factor experiments, Design-Expert 13 software was employed to conduct experimental design, data analysis, and model establishment using the Box–Behnken response surface methodology. The process flow for preparing E-AGP using disodium ethylenediaminetetraacetate was optimized, and the optimal combination of preparation variables was determined (as shown in Table 1). The Box–Behnken design employed in this study comprised 17 experimental points, with all experiments conducted in randomized order.

#### 2.5.2. Response Surface Optimization Validation Experiment

Based on the optimal parameter combinations generated by the response surface methodology, three independent replicate validation experiments were conducted to quantify the repeatability error and systematic deviation in the optimized process.

### 2.6. Sugar Content Testing

The total sugar content of AGP and EAGP was determined using the phenol–sulfuric acid method [25]. The principle involves the hydrolysis of complex carbohydrates in concentrated sulfuric acid to their constituent monosaccharides, followed by further dehydration to form intermediates, which then condense with phenol to yield a yellow aromatic product exhibiting characteristic absorption at 490 nm.

### 2.7. Measurement of Intrinsic Viscosity

The intrinsic viscosity of an optimized 3% (*w*/*v*) EAGP aqueous solution was determined at room temperature using an NDJ-8S rotary viscometer (NDJ-8S, Shanghai Langgan Analytical Instrument Co., Ltd., Shanghai, China) rotary viscometer, following a modified literature protocol [26].

### 2.8. Scanning Electron Microscope (SEM) and Elemental Analysis

The microstructure of EAGP was observed using a SUPRA 55VP field-emission scanning electron microscope (FE-SEM; Carl Zeiss AG, Oberkochen, Germany) scanning electron microscopy (SEM). An appropriate amount of polysaccharide sample was placed on a sample holder, coated with gold using an ion sputter coater, and then examined under a set acceleration voltage to capture images. The elemental composition and distribution were analyzed using an energy dispersive X-ray (EDX) detector (Bruker Nano GmbH, Berlin, Germany) [27].

### 2.9. Fractionation and Purification of EAGP

EAGP was fractionated by ion exchange chromatography on a DEAE-FF (60.6 × 131 mm, GE Healthcare Bio-Sciences AB, Uppsala, Sweden). Samples were separated using water and 0.1 M, 0.2 M, 0.3 M, 0.5 M, and 1.0 M NaCl solutions at a flow rate of 3 mL/min. Elution profiles were monitored using the anthrone–sulfuric acid method. The fraction eluting at the highest yield was further purified using a Superdex-200 gel filtration column (20.6 × 1000 mm, GE Healthcare Bio-Sciences AB, Uppsala, Sweden) with distilled water (flow rate 0.2 mL/min). Elution curves were plotted using the anthrone–sulfuric acid method. The collected homogeneous polysaccharide fraction was lyophilized to obtain purified EAGP-W1.

### 2.10. Monosaccharide Composition Analysis

Monosaccharide composition analysis was performed using the PMP method [28]. Sample Hydrolysis and Derivatization: After drying, add 1 mL of 2 M TFA to the sample and hydrolyze at 121 °C for 2 h. Evaporate the acid solution under nitrogen, wash repeatedly with methanol (2–3 times), then redissolve in sterile water. Take 0.2 mL of the hydrolysate (or standard), sequentially add 0.2 mL of 0.5 M NaOH and 0.5 mL of 0.5 M PMP in methanol, and react at 70 °C for 1 h. After cooling, neutralize with 0.2 mL 0.5 M HCl. Remove residual reagents by extracting with chloroform (1 mL × 3 times). Take the aqueous layer, dilute to the final volume, and proceed for analysis. Chromatography conditions: Thermo U3000 HPLC system with an Agilent ZORBAX Eclipse XDB-C18 column (4.6 × 250 mm, 5 μm, Agilent Technologies, Santa Clara, CA, USA). Mobile phase: acetonitrile: phosphate buffer (12 g/L KH_2_PO_4_, pH 6.8) = 17:83 (*v*/*v*, isocratic elution); flow rate 0.8 mL/min; column temperature 30 °C; detection wavelength 250 nm; injection volume 10 μL.

### 2.11. Methods for Evaluating the Protective Effects of EAGP-W1 in a DSS-Induced Colitis Model

#### 2.11.1. Animal Model and Experimental Groups

Experimental grouping and design reference [29,30], C57BL/6 mice (SPF grade, 8 weeks old, 17–20 g) were acclimatized for one week prior to experimentation in accordance with the Animal Care Committee guidelines. Mice were divided into four groups (*n* = 7): Control, EAGP-W1, DSS, and DSS + EAGP-W1. The preventive treatment regimen involved intragastric administration of EAGP-W1 (100 mg/kg) daily from day 1 to day 10. To induce acute colitis, 3% DSS was added to the drinking water of the respective model groups from day 4 to day 10, while the control groups received distilled water. The Disease Activity Index (DAI) was assessed daily based on body weight loss, stool consistency, and gross bleeding.

#### 2.11.2. Sample Harvesting and Histological Evaluation

Following anesthesia and sacrifice, the spleen and colon were excised. The colon length was measured, and the spleen index was calculated as described previously [31]. Colon specimens were fixed in 4% paraformaldehyde for 24 h, embedded in paraffin, and cut into 3–4 μm sections for hematoxylin and eosin (H&E) staining. To collect cecal contents, the cecum was dissected, and the contents were carefully scraped onto aluminum foil. These samples were then placed in cryovials, flash-frozen in liquid nitrogen, and maintained at −80 °C until analysis.

#### 2.11.3. Gut Microbiota Analysis

Total genomic DNA from stool samples was extracted using the FastPure Stool DNA Isolation Kit. DNA integrity was assessed by 1% agarose gel electrophoresis, and concentration and purity were determined using the NanoDrop 2000 spectrophotometer (Thermo Fisher Scientific, Wilmington, DE, USA). Using the extracted DNA as template, PCR amplification of the 16S rRNA gene V3-V4 region was performed with barcoded primers 338F (5′-ACTCCTACGGGAGGCAGCAG-3′) and 806R (5′-GGACTACHVGGGTWCTTAAT-3′). Libraries were constructed using the NEXTFLEX^®^ Rapid DNA-Seq Kit (MJYH, shanghai, China) and sequenced on the Illumina NextSeq 2000 PE300 platform (https://www.majorbio.com/).

#### 2.11.4. SCFAs Content Determination

SCFA levels in mouse fecal samples from each group were determined using high-performance liquid chromatography (HPLC). The chromatographic system employed an Agilent 8890B-7000D gas chromatography–mass spectrometry (GC-MS) instrument for analysis, equipped with an HP-FFAP capillary column (30 m × 0.25 mm × 0.25 μm) for detection. The SCFA content determination results were analyzed using Agilent MassHunter quantitative software (Agilent, USA, version number: v10.0.707.0) for data acquisition and peak integration. The absolute concentrations of each short-chain fatty acid in the samples were calculated based on the standard curve.

### 2.12. Statistical Analysis

Data were processed using Origin 9.8.0 software, GraphPad Prism 10, and Design-Expert 13. A *p* < 0.05 was considered to be of statistically significant difference. Gut microbiota and short-chain fatty acid analyses were conducted on the Meiji Bio Cloud Platform. Intergroup differences in alpha diversity (Chao1 and Shannon indices) were assessed using the Wilcoxon signed-rank test. Principal coordinate analysis (PCoA) based on Bray–Curtis distances was performed, combined with PERMANOVA testing to evaluate intergroup differences in community structure. LEfSe analysis (LDA > 2, *p* < 0.05) was used to identify significantly different bacterial taxa between groups.

## 3. Results and Discussion

### 3.1. Effects of Individual Factors on the Preparation Process of EDTA-Modified Almond Gum Polysaccharides

#### 3.1.1. Effect of EDTA Concentration

**Figure 1:**
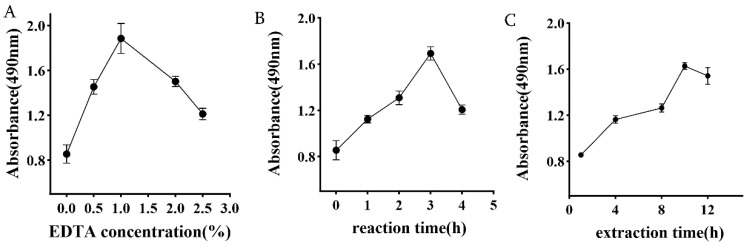
Results of single-factor experiments. (**A**) Effect of EDTA concentration; (**B**) Effect of reaction time; (**C**) Effect of extraction time.

As shown in Figure 1, the absorbance of the phenol–sulfuric acid reaction product exhibits a trend of first increasing and then decreasing with increasing concentration of EDTA. At concentrations below 1%, absorbance increases with concentration, indicating elevated polysaccharide content. This effect effectively chelates metal ions, thereby promoting polysaccharide release. At a 1% concentration, absorbance reaches a peak, signifying saturation of metal ion chelation and maximum measured absorbance, indicating peak polysaccharide content. Beyond 1% concentration, absorbance decreases. This decline may result from excessive EDTA altering the solution’s ionic strength and microenvironment, thereby affecting polysaccharide solubility or stability. The absorbance first increases and then decreases as the EDTA concentration rises. This is related to EDTA’s ability, as a multidentate ligand, to effectively chelate divalent cations (such as Ca^2+^ and Mg^2+^) that play a bridging role in the polysaccharide network. The removal of metal ions disrupts the structure or physical cross-linking network of the polysaccharides, thereby promoting their release and dissolution. However, excessively high concentrations of EDTA can significantly alter the ionic strength of the solution, potentially causing the conformation of polysaccharide molecules to contract or inducing a salting-out effect, thereby reducing their stability in the solvent and the extraction efficiency [13,16]. Therefore, the optimal concentration range of 0.5%, 1%, and 2% disodium ethylenediaminetetraacetate was selected for subsequent experiments.

#### 3.1.2. Impact of Reaction Time

The chelation of EDTA is a chemical reaction process requiring sufficient time for diffusion, contact, and dissociation of the metal ion–polysaccharide complex. When reaction time is less than 2 h, chelation is incomplete, and absorbance increases rapidly over time. When the reaction time reaches 2 h, chelation approaches its peak, absorbance reaches a maximum, and then gradually decreases. Therefore, 2, 3, and 4 h are selected as the optimal reaction time range.

#### 3.1.3. Effect of Extraction Time

As shown in Figure 1, the absorbance increased with time during extraction from 1 to 8 h. However, after 10 h of extraction, the polysaccharide content gradually decreased with increasing extraction time. When the extraction time was insufficient, the polysaccharides failed to dissolve completely. The downward trend in extraction yield over time suggests that while sufficient time facilitates the diffusion of the chelation reaction and the elution of polysaccharide molecules, excessively long reaction times or extraction processes may induce degradation of the polysaccharide backbone or side chains [14]. This degradation disrupts the molecular integrity of the polysaccharides, causing the absorbance to decline after reaching its peak [24]. Therefore, extraction times of 8, 10, and 12 h were selected for subsequent experiments.

### 3.2. Analysis of Response Surface Optimization Experiment Results

#### 3.2.1. Model Building and Significance Tests

This experiment used the Design-Expert 13 software to generate experimental design, statistical analyses, and regression models. Based on single-factor trial results, a three-factor, three-level response surface design was constructed with EDTA concentration (A), reaction time (B), and extraction time (C) as factors. The results are shown in Table 2. The Box–Behnken method was employed to determine the optimal process for preparing EDTA-based almond gum polysaccharides. The optimization experiment randomly selected 17 experimental points, with all experiments replicated three times. The model equation for the response variable is as follows:

*Y* = 1.35208 − 0.119737A − 0.009075B + 0.063437C − 0.085675AB − 0.027500AC − 0.074675BC − 0.228315A^2^ − 0.301940B^2^ − 0.202065C^2^

To determine statistical significance, an f-test and analysis of variance were performed on the quadratic model in the RSM, with results shown in Table 3. For absorbance, the model’s *p*-value was 0.0069, indicating that the model is significant and effectively captures the relationship between the factors and absorbance. The *p*-value for the misfit term exceeded 0.05, indicating non-significance and confirming the model adequately fits the data. Additionally, the *p*-value sequence A < C < B indicates that EDTA exerts the strongest influence. This effect likely arises because an optimal EDTA concentration chelates metal ions, thereby enhancing polysaccharide release and significantly increasing the sugar content of the final product, which in turn leads to higher absorbance values. These results further demonstrate the pivotal role of EDTA in the preparation of almond gum polysaccharides. Since the inherent high viscosity of almond gum is primarily caused by a macroscopic network structure mediated by metal ions, the concentration of the chelating agent becomes the most critical limiting factor in breaking down this network, reducing the system’s viscosity, and increasing the yield [17]. The significance of the quadratic terms (A^2^, B^2^, C^2^) in the model indicates that the relationship between the factors and yield is not a simple linear one, but rather exhibits distinct inflection points, thereby confirming the necessity of using a Box–Behnken design for parameter optimization [18].

#### 3.2.2. Response Surface and Contour Analysis

**Figure 2:**
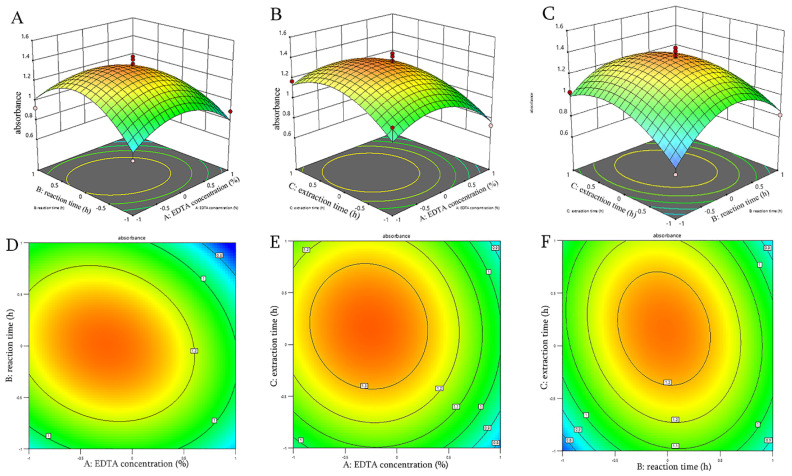
Response surface plots (3D) and contour plots (2D) showing the interactive effects of extraction variables on the absorbance of almond gum polysaccharides. (**A**,**D**) Interaction between EDTA concentration and reaction time; (**B**,**E**) Interaction between EDTA concentration and extraction time; (**C**,**F**) Interaction between reaction time and extraction time. Note: The red dots indicate the max.

Three-dimensional response surface plots and their corresponding contour plots can visually illustrate the effects of interactions between two factors on the response. Typically, the surface’s slope reflects the interaction’s sensitivity; a steeper slope indicates a more pronounced interaction [32]. As shown in Figure 2, the contour lines corresponding to the interaction between Factor A (EDTA concentration) and Factor B (reaction time) form an elliptical shape, exhibiting the steepest gradient among all response surface curves. In contrast, the contour lines for the interactions between B and C, as well as A and C, are closer to circular shapes, with relatively gentler response surfaces. Overall, the influence of interaction terms on polysaccharide content follows the order: AB > BC > AC.

Compared to AGP prepared via conventional water extraction and alcohol precipitation, the EDTA preparation method offers significant advantages, including reduced viscosity and enhanced sugar content. Both methods yield 77–85%, representing an acceptable trade-off. This outcome perfectly aligns with the original process design objective of “addressing the high viscosity and challenging separation/purification of natural gum.” Viscosity decreased from AGP: 640.8 mPa·s to EAGP: 238.7 mPa·s. This reduction signifies the process’s success, indicating that EDTA, as a metal ion chelating agent, effectively sequestered metal ions (e.g., Ca^2+^, Mg^2+^) bound to polysaccharide molecules. Following ion removal, polysaccharide solubility improved, and fluidity increased.

### 3.3. SEM and Elemental Analysis

**Figure 3:**
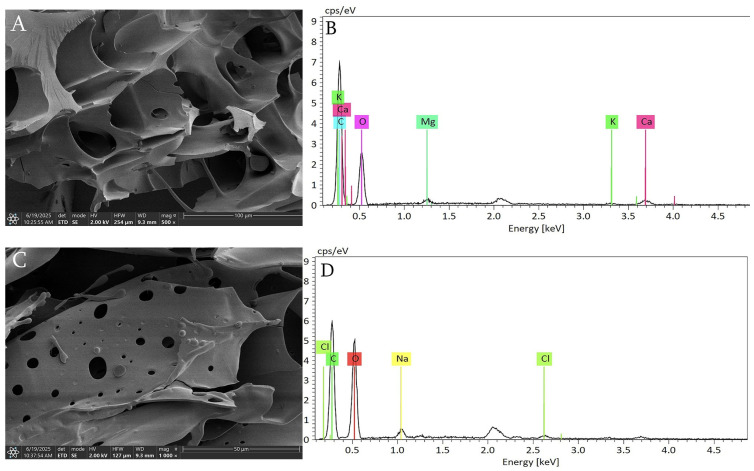
SEM—EDX elemental Analysis. (**A**) SEM Analysis of AGP; (**B**) elemental analysis of AGP-W1; (**C**) SEM Analysis of EAGP; (**D**) elemental analysis of EAGP-W1.

The effects of EDTA treatment on the microstructure and elemental composition of polysaccharides were investigated using SEM-EDX technology. Analysis revealed that AGP particles exhibited smooth and relatively intact surfaces, though their distribution was irregular. EDX analysis showed significant signals from metal ions, including K, Ca, and Mg. These ions likely exist in a bound state within the polysaccharide network, forming “ionic bridges” that increase viscosity [33]. Following EDTA treatment, pores appeared on the surface of EAGP particles, revealing multiple circular openings, while no metal ions were detected. The results indicate that EDTA, acting as a metal-ion chelator, effectively stripped metal ions bound to polysaccharide molecules, disrupting the metal-ion-mediated cross-linking network between polysaccharide molecules and thereby reducing viscosity (Figure 3).

### 3.4. Fractionation and Purification Results of EAGP

**Figure 4:**
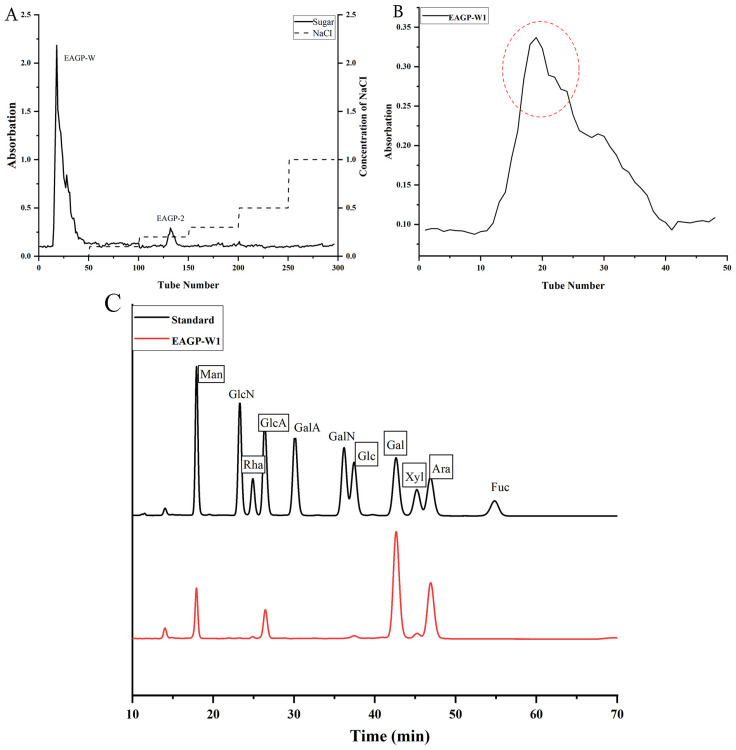
Fractionation and Purification Profile of EAGP; (**A**) Fractionation profile of EAGP; (**B**) Purification elution profile of EAGP-W1 (The red dashed circle indicates the target peak of EAGP-W1 collected for further analysis.); (**C**) Monosaccharide composition analysis of EAGP-W1.

As shown in Figure 4A, EAGP was separated by DEAE-FF chromatography into two fractions (EAGP-W and EAGP-2). The water-eluted fraction yielded a high recovery rate of 38.5%. Following further purification by Superdex-200 gel filtration chromatography, a single fraction, EAGP-W1, was obtained (Figure 4B). Monosaccharide composition analysis (Figure 4C) revealed that EAGP-W1 comprised mannose, rhamnose, glucuronic acid, glucose, galactose, xylose, and arabinose with molar percentages of 7.798, 1.089, 6.741, 1.286, 40.511, 4.193, and 38.382, respectively. High levels of arabinose and galactose are characteristic features of arabinogalactan (AG) or arabinogalactan protein (AGP). The core structure of commercial gum arabic (derived from the acacia tree) consists of a main chain of β-(1→3)-linked galactose with numerous branches composed of arabinose, rhamnose, and other sugars [34,35]. EAGP-W1 exhibits a dominant Gal and Ara content (combined nearly 80%), strongly indicating its classification as an arabinogalactan-type polysaccharide. This aligns with the common structural units found in numerous plant gums, such as peach gum and Tragacanth gum [36,37]. These gum polysaccharides typically feature highly branched structures. If a small number of galacturonic acid residues are located within highly coiled or dense molecular regions, their charge sites may be “shielded” or “enveloped” by surrounding neutral sugar chains (such as arabinose or galactose side chains), hindering contact with the chromatographic column packing. Consequently, these minor galacturonic acid residues fail to adsorb tightly and elute with the water wash.

Notably, the use of EDTA in the preparation of apricot gum polysaccharides not only reduced the intrinsic viscosity but also preserved the essential arabinogalactan core structure, which is known to exhibit high fermentability by beneficial gut bacteria [38,39,40]. Polysaccharides with such physicochemical properties are often recognized as potent prebiotics, capable of selectively promoting the growth of probiotic taxa and generating functional metabolites [41,42]. Therefore, based on the identified structural features and the improved fluidity of EAGP-W1, we hypothesized that it possesses significant prebiotic potential to modulate the intestinal microenvironment and alleviate inflammatory responses. To test this hypothesis, a DSS-induced mouse colitis model was subsequently employed to evaluate the protective efficacy of EAGP-W1 on the ‘microbiota–metabolite–intestinal barrier’ axis.

### 3.5. Evaluation of the Protective Effects of EAGP-W1 in a DSS-Induced Colitis Model

**Figure 5:**
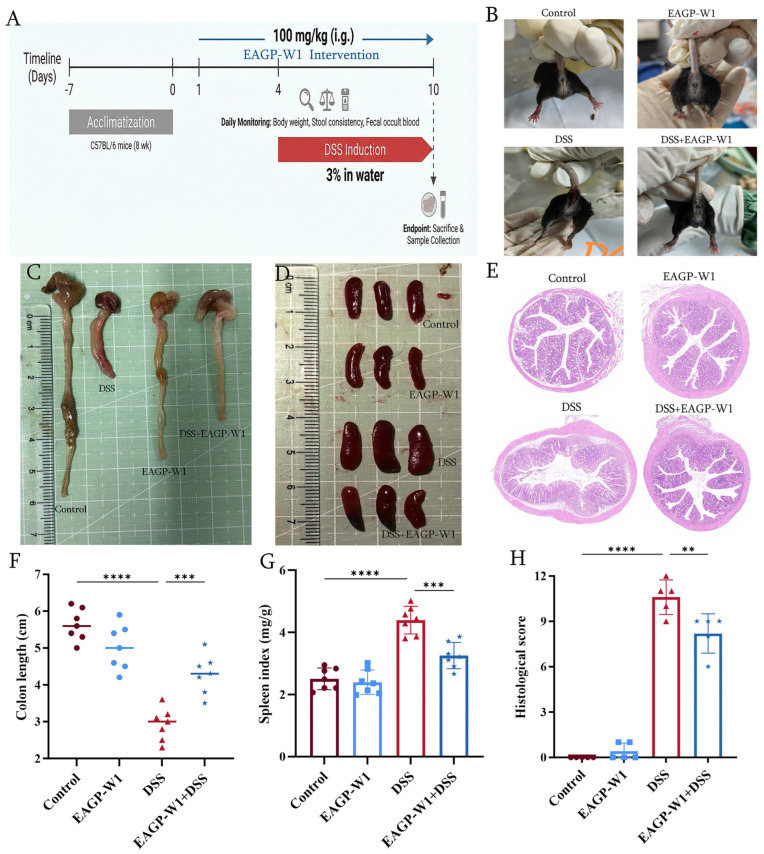
EAGP-W1 relieved DSS caused UC; (**A**) Experimental schedule; (**B**) Representative photos of anuses; (**C**) colons; (**D**) spleens; (**E**) H&E staining of distal colon tissues; (**F**) Colon length; (**G**) Spleen index for the mice; (**H**) Histological score. ((Individual data points are represented by distinct colors and symbols for each experimental group: [Control] (dark red, circles), [EAGP-W1] (light blue, squares), [DSS] (bright red, triangles), and [DSS+EAGP-W1] (dark blue, stars). The height of each bar represents the mean value, and the error bars indicate the [Standard Deviation (SD)/Standard Error of the Mean (SEM)]. The horizontal lines above the bars denote statistically significant differences between the indicated groups. ** and *** indicated *p* < 0.05, **** indicated *p* < 0.01, respectively).

EAGP-W1 shares a composition similar to numerous gum polysaccharides, exhibiting a typical arabinogalactan profile. Recent structure–activity relationship studies confirm that such natural gum polysaccharides not only possess excellent immunomodulatory activity but also effectively alleviate DSS-induced colitis by reshaping the gut microbiota microecology and repairing the intestinal mucosal barrier [21]. Based on the correlation between these compositional characteristics and biological activity, to evaluate the therapeutic potential of EAGP-W1 for ulcerative colitis (UC), we employed the classical dextran sulfate sodium (DSS)-induced method to establish an acute colitis model in mice (Figure 5A).

Observation of mouse anus and fecal status (Figure 5B). Control and EAGP-W1 groups showed normal findings; the DSS group exhibited marked fecal blood and loose stools (perianal soiling); the DSS+EAGP-W1 group demonstrated significantly reduced fecal blood. This reflects EAGP-W1’s ability to alleviate DSS-induced clinical diarrhea and fecal blood symptoms. Colon shortening and spleen enlargement are key macroscopic indicators of colitis severity [43]. The DSS group exhibited extremely significant colon shortening and markedly enlarged spleens; the DSS+EAGP-W1 group showed significant restoration of colon length compared to the DSS group, with reduced spleen enlargement (Figure 5C,D,F,G). These findings indicate that EAGP-W1 effectively inhibits colonic atrophy and reduces systemic inflammatory burden. Histological examination of colonic cross-sections revealed intact mucosal architecture in the Control/EAGP-W1 group. The DSS group exhibited mucosal destruction, crypt loss, and massive infiltration of inflammatory cells. In contrast, the DSS+EAGP-W1 group maintained relatively intact mucosal architecture with preserved crypts (Figure 5E). This histological analysis confirmed EAGP-W1’s restorative and protective effects on the intestine, as well as its ability to suppress inflammatory infiltration. The experimental results demonstrate that EAGP-W1 effectively alleviates DSS-induced ulcerative colitis in mice.

### 3.6. The Role of EAGP-W1 in Reshaping the Gut Microbiota Structure of Colitis Mice

In exploring the intervention mechanisms of EAGP-W1 for ulcerative colitis (UC), maintaining gut microbiota homeostasis is a core research direction. Gut dysbiosis is commonly recognized as a key driver in the development and progression of UC [44,45].

**Figure 6:**
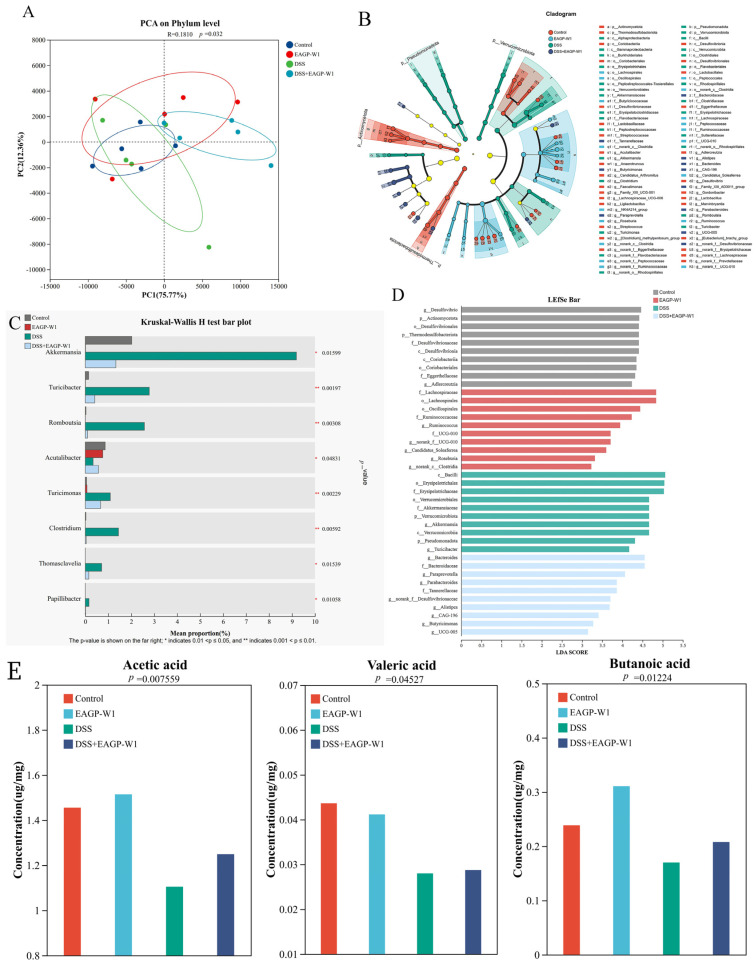
EAGP-W1 reshaped the intestinal bacteria in DSS mice; (**A**) PCA; (**B**) Taxonomic abundance analysis on differentially enriched taxa using LEFSe; (**C**) LDA score; (**D**) Differences in average relative abundance among individuals of the same species; (**E**) Concentration of short-chain fatty acids (acetic acid, valeric acid, and butanoic acid) in mouse feces.

To evaluate the overall remodeling capacity of polysaccharide EAGP-W1 on the intestinal microbiota, we performed principal component analysis (PCA) at the phylum level based on 16S rRNA sequencing data. As shown in Figure 6A, PC1 explained 75.77% of the variance, while PC2 accounted for 12.36%. Statistical analysis revealed *p* = 0.032 (*p* < 0.05) and R = 0.1810, indicating significant differences in gut microbiota structure at the phylum level among the mouse groups. The confidence ellipses for the Control and EAGP-W1 groups largely overlapped with similar distribution areas, indicating that EAGP-W1 had minimal impact on the overall phylum-level structure of normal mouse gut microbiota and did not alter its natural equilibrium. The DSS group samples showed a distinct shift, separating from the Control group. This indicates that DSS-induced colitis significantly altered gut microbiota composition at the phylum level, leading to dysbiosis. In contrast, the EAGP-W1 intervention significantly modulated the microbiota structure at the phylum level in colitis mice, resulting in distinct clustering patterns compared to the model group.

Figure 6B,D illustrates the characteristic microbial differences in the gut microbiota of each mouse group at the taxonomic level. This phylogenetic tree visualizes species differences across various taxonomic levels, providing an intuitive representation of the species that differ between groups at different taxonomic levels. Legend: Nodes of different colors indicate microbial communities that are significantly enriched in their respective groups and have a significant impact on intergroup differences; light yellow nodes indicate microbial communities that show no significant differences across groups or have no significant impact on intergroup differences. In the LEfSe evolutionary branch diagram, the significantly enriched differential species in the Control group were primarily concentrated in Actinomycota (phylum Actinomycota) and its subordinate taxonomic units, maintaining a dominant microbial community structure centered on lactobacilli, reflecting the gut microbiome characteristics under steady-state conditions [46]. In the DSS-induced colitis group, significantly enriched species underwent a fundamental shift, primarily manifested by the explosive growth of Pseudomonadota (Pseudomonadota/Protochlamydota). The marked enrichment of Proteobacteria (which contains multiple opportunistic pathogens) is the most prominent hallmark of dysbiosis [47,48]. The increased abundance of these species directly reflects the selection pressure exerted by the inflammatory intestinal environment on pathogenic bacteria, indicating that DSS severely disrupts the original microbial balance. Akkermansiaceae and Akkermansia within the Eubacteroides phylum are widely recognized as next-generation probiotics [49]. Their marked increase indicates EAGP-W1 possesses potent prebiotic growth-promoting activity. Roseburia, a core butyrate-producing bacterium within Lachnospiraceae, showed significant enrichment in the gut, indicating enhanced short-chain fatty acid production capacity [50]. This result demonstrates that EAGP-W1 functions as a high-quality prebiotic, specifically increasing the abundance of key bacterial genera with barrier repair and anti-inflammatory functions. Compared to the DSS group, theDSS+EAGP-W1 group did not exhibit enrichment of the Proteobacteria phylum. Instead, it shifted toward enriching Bacteroidetes groups with polysaccharide-degrading capabilities and metabolic regulatory functions. Notably, significant increases in Alistipes and Parabacteroides indicated a transition in gut microbiota structure from an inflammatory to a recovery profile [51], suggesting that EAGP-W1 counteracts inflammatory damage by reshaping the microbial community. Differential abundance analysis revealed that the EAGP-W1 intervention significantly enriched functional bacterial groups, including Bacteroides and Alistipes. This confirmed its restorative effect on the gut microbiota of colitis at the taxonomic level, reshaping the gut microbiota structure in DSS-induced colitis mice.

The comparative analysis of relative abundance changes among groups for individual species is shown in Figure 6C. Among the eight core genera detected, the abundance distribution across groups exhibited statistically significant differences (*p* < 0.05 or *p* < 0.01), further quantitatively confirming the restorative effect of polysaccharides on dysbiosis. Combining LEfSe evolutionary branch diagrams with the Kruskal–Wallis H test results yields the following definitive conclusions: DSS-induced colitis manifests not only as macroscopic structural disruption but also as quantitative surges in inflammation-associated genera such as Turicibacter, Romboutsia, and Clostridium. EAGP-W1 intervention directly reverses the expansion of these genera, demonstrating clear anti-inflammatory regulatory potential. The promotion of next-generation beneficial microorganisms Akkermansia by EAGP-W1 is global, significantly increasing its proportion even in non-inflammatory states, providing quantitative evidence for its role in protecting the intestinal mucosal barrier [52]. EAGP-W1’s restoration of the colonitis mouse microbiota is not achieved through a single pathway. Instead, it employs a bidirectional regulatory mechanism: downregulating pro-inflammatory characteristic bacteria while upregulating barrier-protective bacteria, thereby guiding the imbalanced gut microbiome toward a new steady state. Rigorously demonstrated that EAGP-W1 possesses the functions of reshaping the gut microbiota, suppressing the expansion of inflammation-associated bacteria, and specifically proliferating probiotics. This provides a robust microbiological basis for its use as a bioactive molecule in treating ulcerative colitis.

### 3.7. EAGP-W1 Promotes the Generation of SCFAs

SCFAs are primary metabolites produced by gut microbiota through the fermentation of polysaccharides, serving as core indicators for evaluating polysaccharide bioactivity and their role in repairing colitis [53]. Experimental results (Figure 6E) show that the DSS-induced colitis model is accompanied by gut microbiota dysbiosis, with decreased abundance of SCFA-producing bacteria (e.g., butyrate-producing Faecalibacterium and acetate-producing Akkermansia). Reduced SCFA levels may exacerbate intestinal mucosal barrier damage, inflammatory responses, and energy supply insufficiency [54]. E AGP-W1 significantly elevates acetate, butyrate, and valerate levels in the intestines of healthy mice and partially restores SCFA production in DSS-induced colitis models. Butyrate serves as the primary energy source for colonic epithelial cells, exhibiting potent anti-inflammatory properties and enhancing mucosal barrier function [55]. The marked increase in butyrate content by EAGP-W1 directly demonstrates its capacity as an efficient fermentation substrate for butyrate-producing bacteria (such as Roseburia, as identified in the aforementioned LEfSe analysis). Although SCFA levels in the DSS+EAGP-W1 group were lower than those of the control group, they were significantly higher than those of the DSS-only group, indicating that EAGP-W1 retains some gut microbiota regulatory effects even under inflammatory conditions. The extent of recovery may be related to the fermentation characteristics of polysaccharides, the duration of intervention, or the capacity for microbiota restoration. The findings suggest that EAGP-W1 may serve as a fermentation substrate for specific probiotics (e.g., Bifidobacterium, Lactobacillus, or butyrate-producing bacteria), thereby promoting SCFA synthesis and effectively reversing DSS-induced downregulation of intestinal SCFAs (particularly acetate and butyrate). This metabolic restoration directly results from polysaccharides reshaping the gut microbiota, providing the crucial biochemical basis for EAGP-W1’s ability to alleviate colitis and protect the intestinal barrier.

## 4. Conclusions

This study successfully established an optimized EDTA-assisted extraction process for almond gum polysaccharides, effectively overcoming the industrial bottleneck of high viscosity. Under the identified optimal conditions (1% EDTA concentration, 3 h reaction time, and 10 h extraction time), the intrinsic viscosity of the polysaccharide was significantly reduced from 640.8 mPa·s to 238.7 mPa·s, substantially improving its processing fluidity. Structural characterization revealed that the purified fraction, EAGP-W1, is a branched arabinogalactan-type polysaccharide primarily composed of galactose (40.51%) and arabinose (38.38%) with a porous microstructure. In the DSS-induced mouse model, EAGP-W1 demonstrated potential protective effects against colitis by alleviating colon shortening and histological damage. These effects were closely associated with the modulation of the gut microbiota, specifically the enrichment of beneficial genera such as Akkermansia and Roseburia, which in turn promoted the production of total short-chain fatty acids (SCFAs).

The physicochemical transformation of almond gum via EDTA modification opens new avenues for industrial applications. Firstly, the significantly reduced viscosity enables EAGP to serve as a high-performance hydrocolloid or processing aid. Unlike the extremely viscous natural gum, which limits its addition level, EAGP can be seamlessly incorporated into beverages and liquid formulations without negatively impacting the sensory profile or texture. Secondly, the identification of EAGP-W1 as an arabinogalactan similar to commercial Gum Arabic highlights its potential as a premium prebiotic polysaccharide. Its specific ability to promote SCFA-producing bacteria positions it as a valuable candidate for the development of synbiotic food products. Lastly, as a novel functional ingredient, EAGP-W1 offers a dual benefit: it imparts structural stability to food matrices while delivering targeted protective effects against intestinal inflammatory stressors. Ultimately, these findings establish a robust scientific basis for its utilization in dietary supplements aimed at gut health maintenance.

## Figures and Tables

**Table 1:** Factors and levels for response surface analysis.

Level	Factors		
	EDTA Concentration (%)	Reaction Time (h)	Extraction Time (h)
−1	0.5	2	8
0	1	3	10
1	2	4	12

**Table 2:** Experimental results of response surface optimization design.

Experiment Number	Independent Variable			Dependent Variable
	EDTA Concentration (%)	Reaction Time (h)	Extraction Time (h)	Absorbance (nm)
1	−1	−1	0	0.7896
2	0	0	0	1.4141
3	0	−1	1	1.0311
4	0	1	1	0.8821
5	−1	0	−1	1.0802
6	−1	1	0	0.9243
7	0	1	−1	0.8144
8	0	0	0	1.3722
9	1	−1	0	0.8907
10	0	−1	−1	0.6647
11	−1	0	1	1.1719
12	0	0	0	1.4439
13	0	0	1	0.7082
14	0	0	0	1.3222
15	1	0	−1	0.7265
16	0	0	0	1.208
17	1	1	0	0.6827

**Table 3:** ANOVA for Response Surface Quadratic Model Analysis.

Source	Sum of Squares	df	Mean Squares	F-Value	*p*-Value
Model	1.06	9	0.1183	7.63	0.0069
EDTA concentration	0.1147	1	0.1147	7.40	0.0298
B- reaction time	0.0007	1	0.0007	0.0425	0.8426
C- extraction time	0.0322	1	0.0322	2.08	0.1928
AB	0.0294	1	0.0294	1.89	0.2112
AC	0.0030	1	0.0030	0.1951	0.6721
BC	0.0223	1	0.0223	1.44	0.2694
A^2^	0.2195	1	0.2195	14.15	0.0071
B^2^	0.3839	1	0.3839	24.75	0.0016
C^2^	0.1719	1	0.1719	11.09	0.0126
Residual	0.1086	7	0.0155		
Lack of Fit	0.0742	3	0.0247	2.88	0.1664
Pure Emor	0.0343	4	0.0086		
Cor Total	1.17	16			
R^2^ = 0.9075					

## Data Availability

The original contributions presented in the study are included in the article. Further inquiries can be directed to the corresponding author.

## Abbreviations

EDTA	Ethylenediaminetetraacetic acid
AGP	Almond gum polysaccharides
EAGP	EDTA-soluble polysaccharides from almond gum
EAGP-W1	Purified fraction of EAGP
DSS	Dextran sulfate sodium
SCFAs	Short-chain fatty acids
TFA	Trifluoroacetic acid
PMP	1-phenyl-3-methyl-5-pyrazolone
RSM	Response Surface Methodology
BBD	Box–Behnken design
SEM	Scanning electron microscope
EDX	Energy-dispersive X-ray detector
HPLC	High-performance liquid chromatography
GC-MS	Gas chromatography–mass spectrometry
PCR	Polymerase chain reaction
rRNA	Ribosomal ribonucleic acid
DAI	Disease Activity Index
H&E	Hematoxylin and eosin staining
UC	Ulcerative colitis
IBD	Inflammatory bowel disease
SPF	Specific pathogen-free
PCA	Principal component analysis
ANOVA	Analysis of variance
LEfSe	Linear discriminant analysis effect size
LDA	Linear discriminant analysis
